# Pharmacokinetics of Ilunocitinib, a New Janus Kinase Inhibitor, in Dogs

**DOI:** 10.1111/jvp.70022

**Published:** 2025-09-08

**Authors:** Kirsten Boerngen, Yogini Patel, Melissa Pittorino, Céline E. Toutain

**Affiliations:** ^1^ Elanco Animal Health GmbH Monheim am Rhein Germany; ^2^ Elanco Animal Health, Yarrandoo R&D Centre Kemps Creek New South Wales Australia; ^3^ Elanco Animal Health Sèvres France

**Keywords:** canine atopic dermatitis, dog, ilunocitinib, JAK inhibitor, pharmacokinetics

## Abstract

Ilunocitinib, a novel Janus kinase inhibitor, is indicated for managing pruritus and skin lesions associated with canine allergic and atopic dermatitis. Pharmacokinetics of ilunocitinib were investigated following single intravenous and oral administrations, both in fed and fasted states. Dose proportionality was assessed using oral doses ranging from 0.4 to 4.0 mg/kg, and multiple dosing was evaluated with daily oral doses of 0.8 mg/kg. Serial blood samples were collected, and plasma concentrations of ilunocitinib were measured using a validated LC–MS/MS method. Pharmacokinetic samples were also collected in field trials. Intravenous administration resulted in low plasma clearance (0.437 L/h/kg), a volume of distribution of 1.58 L/kg, and a terminal half‐life of 4.4 h. Oral administration led to rapid absorption (*T*_max_ usually ranging between 1 and 4 h) and higher bioavailability in fed dogs (80%) compared to fasted dogs (61%). The prandial effect observed in laboratory studies with single doses was not clinically relevant under field conditions. Exposure increased less than proportionally with increasing doses. No clinically relevant accumulation was observed with 0.8 mg/kg daily dosing. No sex‐based differences were observed. Altogether, ilunocitinib pharmacokinetics support a once‐daily oral dosing in dogs. Minimal accumulation, also confirmed in long‐term studies, further supports the safety of ilunocitinib with a daily dosing regimen.

## 1 Introduction

Canine atopic and allergic dermatitis (cAD), a frequent reason for veterinary consultation, significantly impacts the quality of life for both dogs and their owners (Linek and Favrot 2010). The complex pathogenesis of cAD involves inflammatory and allergic immune responses mediated by various cytokines, many of which signal through the Janus kinase‐signal transducer and activator of transcription (JAK–STAT) pathway (Datsi et al. 2021; Gonzales et al. 2013; Bachmann et al. 2018; Marsella et al. 2018; Chaudhary et al. 2019). This chronic and inflammatory skin disease is commonly seen by general practitioners and accounts for a considerable percentage of cases presented to dermatologists in referral practices (Hillier and Griffin 2001; Drechsler et al. 2024). It involves a combination of genetic predisposition, skin barrier dysfunction, microbial imbalance, immune dysregulation, and allergen sensitization (Favrot et al. 2020). The resulting pruritus further exacerbates cAD by compromising the skin barrier, perpetuating a cycle of inflammation and allergen penetration. Therefore, effective pruritus and inflammatory management are crucial not only for symptomatic relief but also for controlling the underlying disease process.

As a lifelong condition, cAD currently has no cure. While environmental and dietary modifications can contribute to cAD management, targeted pharmacologic intervention remains essential for controlling the persistent and often worsening pruritus and inflammation. Ilunocitinib, a novel Janus kinase inhibitor (JAKi) marketed as Zenrelia™, is indicated for managing pruritus and cAD at the therapeutic dose of 0.6–0.8 mg/kg. This manuscript describes the pharmacokinetic profile of ilunocitinib in dogs, including oral bioavailability, the influence of food, dose proportionality, the effects of repeated administration, and pharmacokinetics under field conditions.

## 2 Materials and Methods

### 2.1 Guidelines, Animal Welfare and Regulatory Standards

The three laboratory studies presented in this manuscript were conducted according to the Guidelines for the conduct of pharmacokinetic studies in target animal species (EMA 2024). Two of those studies were performed at Elanco's test facility located in Australia, in compliance with the Australian code for the care and use of animals (National Health and Medical Research Council 2013). They were conducted under the Australian Pesticides and Veterinary Medicines Authority (APVMA) Small‐scale Trials Permit PER7250, and protocols were reviewed and approved by the test facility Animal Ethics Committee. The remaining study was performed by a Contract Research Organization located in the US in compliance with the Animal Welfare Act Regulations (Code of Federal Regulations 2011, National Research Council 2011). The protocol was reviewed and approved by the Testing Facility Institutional Animal Care and Use Committee.

The Good Laboratory Practice (GLP) studies were performed in compliance with the OECD Principles of Good Laboratory Practice (OECD 1998) and U.S. Food and Drug Administration Good Laboratory Practice for Nonclinical Studies (U.S. Food and Drug Administration, n.d.). The clinical trials were performed according to Good Clinical Practices (GCP) (VICH 2000).

The analytical method was validated according to the US and EU Bioanalytical method validation guidelines (US FDA 2018; EMA 2012).

### 2.2 Treatment and Administration

Commercial tablet formulation of ilunocitinib, available in 4.8, 6.4, 8.5, and 15 mg strengths, was dispensed in all studies presented in this manuscript. Tablets were administered orally to each dog based on their most recent body weight, targeting doses of 0.4, 0.8, 1.6, 2.4, or 4.0 mg/kg, as specified for each study. The tablets were combined and/or halved appropriately to meet the individual's precise dosage requirement. Approximately 5 mL of water was given after tablet administration, and the mouth was checked to ensure the tablets had been swallowed. For intravenous administration, ilunocitinib was dosed as a bolus injection of a 10 mg/mL solution via catheter into the cephalic vein. The volume used was based on the most recent body weight to meet a target dose of 0.8 mg/kg. The catheter was flushed with approximately 1 mL saline after injection.

In order to achieve the fed state, the dogs were fasted overnight (at least 16 h) prior to receiving canned food on the day of dose administration. Animals were offered approximately 200 g of a commercially available canned dog food (Advance Puppy Plus with Lamb and Rice or Adult All Breed Casserole with Chicken, Mars, Australia) 20–30 min prior to dosing. To achieve a fasted state, the dogs were fasted overnight for at least 16 h prior to the time of dosing. On the day of dose administration, the dogs did not receive any food prior to dosing, and they were offered their daily dry ration (Advance Adult All Breed Lamb, Mars Australia, or Certified Canine Diet #5007, PMI Inc.) following the 4‐h post‐dose blood collection.

### 2.3 Bioavailability and Prandial Effect

Sixteen Beagle dogs (eight males and eight females from the test facility's dog colony) aged 15 months to 4 years old and weighing 9 to 17 kg were included in this GLP study and randomized by sex and body weight to treatment groups. During the first and second week of the study, all 16 dogs were treated orally in a crossover design in fed or fasted state with the ilunocitinib tablets at a target dose of 0.8 mg/kg. In the third week of the study, eight dogs (four males and four females), randomly selected from the 16 dogs above, were treated intravenously at a target dose of 0.8 mg/kg in fasted state with a 10 mg ilunocitinib/mL solution as a bolus in the cephalic vein at a target dose rate of 0.8 mg/kg. Each treatment was separated by a 1‐week washout period.

For the oral treatments, blood samples were collected pre‐treatment and post‐treatment at 15, 30, and 45 min, 1, 1.5, 2, 3, 4, 8, 12, 24, 36, 48, and 72 h. For the intravenous treatment, blood samples were collected pre‐treatment and post‐treatment at 2, 10, 15, 30, and 45 min and 1, 1.5, 2, 3, 4, 8, 12, 24, 36, 48, and 72 h.

### 2.4 Dose Proportionality and Repeated Administration

In the first GLP study investigating dose linearity in the fasted prandial state, 25 male Beagle dogs (from the test facility's dog colony) aged 13 months and weighing 8–11 kg were included and randomized by bodyweight to five treatment groups (i.e., 5 dogs per group). Each dog received a single oral administration of the final tablet formulation at target doses of 0.4, 0.8, 1.6, 2.4, or 4.0 mg/kg in a fasted condition on study day 0. In addition, the 0.8 mg/kg dose group received repeated daily administration from study day 3 to 13. Blood samples were collected from all animals following the single dose at pre‐dose and at 0.5, 1, 1.5, 2, 3, 4, 6, 8, 12, 24, 36, 48, and 72 h post‐dose. In addition, for the 0.8 mg/kg dose group, blood samples were also collected at the same time points following repeated dosing on study day 13.

In another non‐GLP study investigating dose linearity in the fed prandial state, 24 Beagle dogs (11 males and 13 females from the test facility's dog colony) aged 13 months to 4 years and 7 months old and weighing 9 to 18 kg were included and randomized by sex and bodyweight to three treatment groups (i.e., 8 dogs per group). Each dog received a single oral administration of the final tablet formulation at target doses of 0.8, 2.4, or 4.0 mg/kg in fed conditions on study day 0. In addition, the 0.8 mg/kg dose group received a single oral administration of the final tablet formulation in fasted conditions on study day 7. Blood samples were collected pre‐treatment and post‐treatment at 15 and 30 min, 1, 2, 3, 5, 8, 24, 36, and 48 h.

### 2.5 Pharmacokinetics in Clinical Trials

Plasma ilunocitinib concentrations were monitored in three clinical studies where ilunocitinib was administered at the therapeutic dose of 0.6–0.8 mg/kg. Clinical study 1 (a positive‐controlled study for pruritus and skin lesion control; Forster, Boegel, et al. 2025) collected single blood samples on study days 28 and 56. Clinical study 2 (a placebo‐controlled study for atopic dermatitis control; Forster, Trout, et al. 2025b) collected samples on study days 28 and 112. Clinical study 3 (a placebo‐controlled study for control of allergic dermatitis; Forster, Trout, et al. 2025a) collected samples on study days 7 and 28. Blood collection, dosing, and most recent feeding times were recorded. Treatment administration was categorized based on the dog's feeding status (with or without food). A dog was considered ‘fed’ if it received at least one third of a regular meal within approximately 30 min prior to dosing; otherwise, it was considered ‘fasted’.

### 2.6 Bioanalysis

Plasma samples were analyzed to determine concentrations of ilunocitinib using a validated method. The internal standard (400 μL of 50 ng/mL baricitinib in 0.2% formic acid in acetonitrile) was added to a Phenomenex Phree phospholipid removal plate. A 100 μL aliquot of standard, quality control samples, or study plasma sample was added to the Phree plate and vortex mixed at 500 rpm for 1 min. The sample extracts were drawn into a 96‐well collection plate using vacuum pressure. After the sample eluent was collected, an additional 1 mL of deionized water was added, the collection plate was sealed, and vortex mixed prior to analysis via LC–MS/MS.

A known volume of the processed sample was injected onto a Phenomenex Kinetex Biphenyl 50 × 2.1 mm, 2.6 μm HPLC column. The samples were eluted at a flow rate of 400 μL/min using a gradient method. Mobile phase A consisted of 0.2% formic acid in deionized water, and mobile phase B was 0.2% formic acid in acetonitrile. The gradient started at 90% A/10% B, shifted to 70% A/30% B over 3.0 min, then moved to 100% B, held from 3.70 to 4.00 min, before reverting to the initial conditions. The analysis run time was 5.0 min.

Detection was performed on a Sciex 6500 Q‐Trap mass spectrometer using Turbo Ion Spray in positive ion mode, monitoring transitions at 384.1 → 251.1 amu and 384.1 → 186.2 amu for ilunocitinib, and 372.0 → 250.9 amu for baricitinib. The retention times were approximately 2.3 min for ilunocitinib and 2.0 min for baricitinib.

Quantification was conducted using the peak area ratio of analyte/internal standard with linear regression and 1/*x* ^2^ weighting, covering a concentration range of 0.500–500 ng/mL. Each analytical run included duplicate standard curves as well as quality control samples (at least 6 sets) with concentrations of 0.500, 1.50, 25.0, 400, and 4000 ng/mL. Chromatograms were integrated and processed using Analyst Software (v1.6.3).

Intra‐run accuracy and precision for single ion transitions ranged from 98.0% to 109% and 4.94% to 11.5% respectively, and for sum transitions it ranged from 99.1% to 114% and 5.01% to 11.0% respectively. Inter‐run variability was minimal, confirming the method's robustness. Samples with concentrations exceeding the upper quantitation limit were re‐assayed after dilution with blank dog plasma. The method was rigorously validated for accuracy, precision, and carryover, ensuring it is reliable for routine ilunocitinib analysis in dog plasma.

### 2.7 Pharmacokinetic Analysis

Pharmacokinetic parameters were calculated from individual concentration vs time profiles using a non‐compartmental analysis (NCA) with the software Phoenix WinNonlin (version 8.6; Certara, Princeton, NJ, USA). The maximum plasma concentration (*C*_max_), time to maximum concentration (*T*_max_), the area under the plasma concentration versus time profile (AUC) from 0 to 24 h (AUC_0‐24_), AUC to infinity (AUC_inf_), and terminal half‐life (*T*_½_) were determined for all animals. *C*_max_ and *T*_max_ were observed values. The AUC was determined by the trapezoidal method using the linear trapezoidal rule to compute AUCs any time that the concentration data was increasing and the logarithmic trapezoidal rule any time that the concentration data was decreasing (i.e., linear up log down). The extrapolated portion of the AUC was calculated using the slope of the log‐linear phase. The terminal half‐life was calculated based on the elimination rate constant that included the final three to four quantifiable concentration‐time points for each subject (*T*_1/2_ = ln(2)/slope). After intravenous (IV) administration, the concentration at time 0 (*C*_0_) was determined for each animal by back extrapolating from a linear regression of the first two time points. The total plasma clearance (CL) (CL = Dose/AUC_inf_) and the volume of distribution at steady‐state (*V*ss) (*V*ss = CL × MRT) were also calculated. Dose normalized PK parameters scaled to a dose of 1 mg/kg were included to derive a consistent base to compare values resulting from experiments with different dose levels. Dose normalization was performed by dividing the concentration or AUC by the individual dose (*D*) in mg/kg. Values that were below the lower limit of quantitation (LLOQ) were set as missing for the pharmacokinetic analysis. Actual dose and nominal timepoints were used for the calculations.

For graphical presentation, geometric mean values from individual plasma concentrations were displayed over the dosing interval of 24 h together with their respective standard deviation. Box plots are displayed on the original scale and were created to compare exposure in terms of AUC_0‐24_. Each box goes from the lower quartile to the upper quartile, with a horizontal line at the mean and a horizontal dashed line at the median; whiskers extend from the minimum to the maximum.

The absolute (intravenous vs. fed and fasted oral routes) and relative (fed vs. fasted) oral bioavailability were calculated using the Bioequivalence module of Phoenix WinNonlin (version 8.6, Certara, Princeton, NJ, USA). A linear mixed‐effects model was fitted to the log‐transformed, dose‐normalized AUC_inf_ values obtained from the non‐compartmental analysis from the eight dogs that received both oral and intravenous administration. The model was specified with the route of administration (intravenous, oral fed, oral fasted) as a fixed effect and the dog as a random effect to account for inter‐subject variability.

The absolute oral bioavailability was calculated by comparing the dose‐normalized AUC_inf_ from each oral route to the intravenous route, which was set as the reference. For the relative bioavailability, the fasted prandial state was compared against the fed state, which was set as the reference to quantify the effect of food on drug absorption. For all comparisons, the geometric least squares mean ratios and their corresponding 90% confidence intervals (CIs) were calculated.

Similarly, exposure in males and females was compared for each route of administration with sex as a fixed effect and males set as the reference. The geometric least squares mean ratio of female to male exposure and its corresponding 90% CI were calculated. A potential sex effect was considered statistically significant if the *p*‐value was less than 0.05.

The relationship between dose and AUC was evaluated to assess dose linearity and proportionality using a hierarchical regression analysis with Phoenix software. First, a quadratic model (AUC = A0 + A1 × Dose + A2 × Dose^2^) was fitted to the data. Dose linearity was accepted if the 95% CI for the quadratic term (A2) contained zero, indicating no significant curvature. If linearity was established, a simpler linear model (AUC = Intercept + Slope × Dose) was then evaluated. Dose proportionality was concluded if the 95% CI for the intercept of this linear model also contained zero.

## 3 Results

### 3.1 Bioavailability and Prandial Effect

**FIGURE 1:**
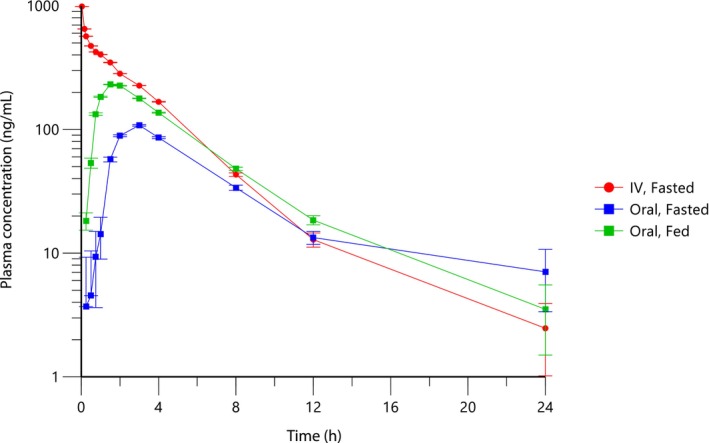
Plasma concentrations (geometric mean ± geometric SD) of ilunocitinib following a single intravenous administration of a solution at 0.8 mg/kg or oral administration of the tablet at 0.8 mg/kg in fed or fasted beagle dogs.

Plasma concentration‐time profiles of ilunocitinib following intravenous and oral administration (0.8 mg/kg) in fed and fasted dogs are shown in Figure 1, with corresponding pharmacokinetic parameters presented in Table 1.

**TABLE 1:** Summary of pharmacokinetic parameters of ilunocitinib following a single intravenous or single oral administration at 0.8 mg/kg in fed or fasted dogs.

Variable (units)	Oral tablet		Intravenous
	Fed 0.8 mg/kg	Fasted 0.8 mg/kg	0.8 mg/kg
	Geo. Mean (CV%)^a^ (*N* = 16)		Geo. Mean (CV%)^a^ (*N* = 8)
*T*_max_ (h)	1.75 (1.0–4.0)	3.0 (0.75–4.0)	NC
*C*_0_ (ng/mL)	NC	NC	1100 (11.1)
*C*_max_ (ng/mL)	268 (32.8)	122 (49.1)	NC
*C*_max_/D (ng/mL)	337 (32.9)	153 (50.3)	NC
AUC_0‐24_ (h*ng/mL)	1290 (23.3)	779 (39.2)	1890 (15.6)
AUC_0‐24_/D (h*ng/mL)	1620 (24.4)	978 (40.0)	2270 (13.7)
AUC_inf_ (h*ng/mL)	1320 (23.6)	913 (35.5)	1910 (15.4)
AUC_inf_/D (h*ng/mL)	1650 (24.7)	1150 (36.2)	2290 (13.5)
%AUC_extra_ (%)	0.397 (63.7)	0.869 (84.7)	0.355 (42.8)
*T*_1/2_ (h)	5.02 (24.2)	5.42 (37.9)	4.42 (27.2)
CL (L/h/kg)	NC	NC	0.437 (13.5)
*V*_ss_ (L/kg)	NC	NC	1.58 (16.1)
*F*_abs_ ^b^ (%)	80 (68–93)	61 (52–72)	NC

Abbreviation: NC, not calculated.

^a^ Geometric mean and (geometric CV%) except for Tmax where the median and (range) are given.

^b^ Absolute bioavailability Fabs was calculated in the eight dogs that received the IV administration, and (confidence interval) is given.

Following intravenous bolus administration, ilunocitinib concentrations declined in a multiphasic manner with a mean terminal half‐life (T_1/2_) of 4.4 h. Plasma clearance (CL) was 0.437 L/h/kg (7.3 mL/min/kg), and the volume of distribution (*V*_SS_) was 1.58 L/kg. Less than 1% extrapolated AUC (%AUC_extra_) indicated that the sampling schedule was sufficient to characterize the concentration‐time profiles. After oral administration, *T*_max_ usually ranged between 1 and 4 h, and terminal *T*_1/2_ was approximately 5 h. Oral administration of ilunocitinib tablets resulted in higher exposure in fed compared to fasted animals.

The statistical analysis showed a significant effect of the route of administration on the total exposure AUC_inf_ (*p* = 0.0004). When administered to fasted dogs, ilunocitinib had an absolute bioavailability of 60.8% (90% CI: 51.6%–71.5%). When administered to fed dogs, the absolute bioavailability increased to 79.6% (90% CI: 67.6%–93.6%).

A comparison of fed and fasted states showed that exposure in fasted dogs was 76.4% of that in fed dogs (90% CI: 64.9%–89.9%). As this is outside the 80%–125% bioequivalence limits, this confirms that co‐administration with food increases oral bioavailability (*p* = 0.0112).

**FIGURE 2:**
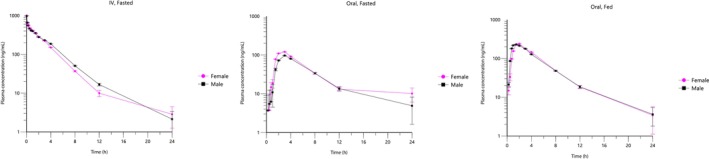
Plasma concentrations (geometric mean ± geometric SD) of ilunocitinib following intravenous and oral administration (0.8 mg/kg) in male or female dogs.

No statistically significant differences in drug exposure were observed between male and female dogs for any administration route (*p* > 0.25 for all comparisons; Figure 2). The geometric mean exposure in females relative to males was 92.2% following intravenous administration (90% CI: 76.1%–111.8%) and nearly identical at 102.3% in the oral fed state (90% CI: 82.2%–127.3%). While the ratio in the oral fasted state was 125.4%, this estimate was associated with high variability (90% CI: 89.7%–175.3%). Overall, sex was not identified as a significant factor influencing the pharmacokinetics of the compound in this study.

### 3.2 Dose Effect in Fasted and Fed Conditions

**FIGURE 3:**
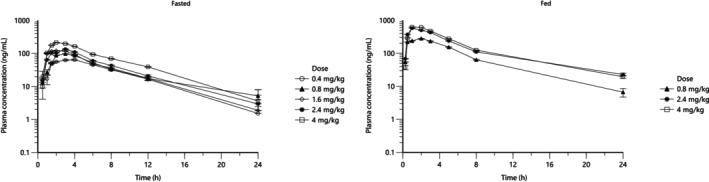
Plasma concentrations (geometric mean ± geometric SD) of ilunocitinib following single oral administration of the tablet in fasted or fed beagle dogs at 0.4, 0.8, 1.6, 2.4 or 4 mg/kg.

Plasma concentration‐time profiles of ilunocitinib following oral administration of 0.4, 0.8, 1.6, 2.4, and 4 mg/kg in fasted conditions and 0.8, 2.4, and 4 mg/kg in fed conditions are presented in Figure 3. Pharmacokinetic parameters thereof are summarized in Table 2.

**TABLE 2:** Summary of PK parameters of ilunocitinib following single administrations of the final tablet at different dose levels in fasted and fed dogs.

Variable (units)	Fasted					Fed			Fasted
	0.4 mg/kg	0.8 mg/kg	1.6 mg/kg	2.4 mg/kg	4.0 mg/kg	0.8 mg/kg	2.4 mg/kg	4 mg/kg	0.8 mg/kg
	Geometric mean (CV%)^a^ (*N* = 5)					Geometric mean (CV%)^a^ (*N* = 8)^c^			
T_max_ (h)	3 (1.5–4)	3 (1–4)	2 (1.5–3)	3 (3–4)	2 (1.5–3)	1.5 (0.5–5)	1 (0.5–2)	1 (0.5–2)	2 (1–3)
C_max_ (ng/mL)	70.8 (95.2)	108 (83.5)	138 (21.9)	136 (24.2)	233 (58.8)	332 (26.3)	605 (30.3)	674 (18.0)	149 (140)
C_max_/D^b^ (ng/mL)	170 (106)	129 (75.0)	81.6 (32.8)	55.1 (23.9)	60.2 (49.4)	416 (28.2)	246 (30.0)	171 (17.3)	187 (134)
AUC_0‐24_ (h*ng/mL)	562 (58.8)	745 (43.0)	831 (35.6)	915 (12.8)	1420 (37.0)	1850 (24.8)	3420 (27.8)	3740 (29.8)	981 (99.7)
AUC_0‐24_/D^b^ (h*ng/mL)	1350 (68.2)	894 (33.9)	493 (40.0)	371 (12.3)	368 (26.9)	2320 (26.0)	1390 (26.2)	948 (30.0)	1230 (95.7)
AUC_inf_ (h*ng/mL)	573 (57.1)	786 (39.7)	860 (38.9)	958 (13.9)	1440 (37.1)	1830 (22.7)	3650 (31.7)	3900 (30.5)	1070 (94.2)
AUC_inf_/D^b^ (h*ng/mL)	1380 (66.4)	942 (30.5)	510 (43.5)	388 (15.7)	373 (27.0)	2290 (24.9)	1490 (30.0)	990 (30.6)	1330 (90.6)
T_1/2_ (h)	3.66 (19.9)	4.29 (51.8)	3.64 (45.0)	4.24 (47.9)	3.73 (10.4)	4.33 (20.9)	5.12 (32.3)	5.04 (20.6)	4.78 (22.6)

^a^ Geometric mean (and geometric CV%) except for *T*_max_ where the median and (range) is given.

^b^ Parameters dose‐normalized to 1 mg/kg.

^c^ Except for AUCinf, AUCinf/D, and T1/2 where *N* = 7 for the 0.8 mg/kg dose level in fed conditions.

In fasted dogs, compared to fed dogs, exposure, as assessed by *C*_max_, AUC_0‐24_, and AUC_inf_ values, showed higher inter‐individual variability across the five dose groups (CV% > 30%). In fed dogs, *C*_max_ and AUC values displayed lower inter‐individual variability (CV% < 30%) compared to fasted dogs.

In the fasted state, the quadratic term A2 was not statistically significant, as its 95% confidence interval (−77.9 to 114.3) contained zero. This indicated that a linear model was the most appropriate fit for the data. The intercept of the subsequent linear model was 332.9, with a 95% confidence interval (189.1to 476.6) that did not include zero. A similar result was observed in the fed state, where the quadratic term was also found to be not statistically significant (95% CI: −505.7 to 71.1). The corresponding linear model for the fed data yielded an intercept of 1313.4, which was also statistically significant (95% CI: 831.3 to 1795.6). Therefore, it was concluded that while the relationship between dose and AUC is linear under both fed and fasted conditions, it is not dose‐proportional due to the significant non‐zero intercepts.

**FIGURE 4:**
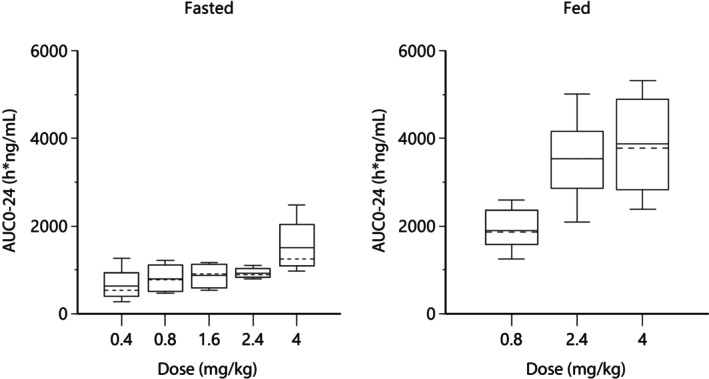
Boxplots of AUCs of ilunocitinib following single oral administration of the tablet in fasted or fed beagle dogs at 0.4, 0.8, 1.6, 2.4 or 4 mg/kg.

In summary and after visual inspection of the data, exposure in a fasted state increased with the increase in target dose level from 0.4 to 4 mg/kg/day (Figure 4). The increases in *C*_max_ and AUC values were less than dose proportional from 0.4 to 2.4 mg/kg and roughly dose proportional between 2.4 and 4.0 mg/kg, as shown by dose‐normalized parameters.

Exposure in the fed state also increased with an increase in dose level from 0.8 to 4.0 mg/kg, and this increase was less than dose proportional.

In addition, the 0.8 mg/kg dose group that received another single oral administration of the final tablet formulation in fasted conditions on study day 7 displayed lower exposure and confirmed the results of the previous study.

### 3.3 Repeated Administration

Exposure, as assessed by ilunocitinib geometric mean *C*_max_, and AUC_0‐24_ was also investigated upon multiple daily administrations at the target dose of 0.8 mg/kg for 11 consecutive days in fasted dogs. Comparison of dose‐normalized AUC_0‐24_ and *C*_max_ values between single and multiple administration (Table 3) resulted in very comparable values in the majority of dogs. However, one dog exhibited a high accumulation ratio (5.66 for *C*_max_/D and 3.39 for AUC_0‐24_/*D*), which influenced the geometric mean accumulation ratios (1.26 for AUC_0‐24_ and 1.45 for *C*max). This dog exhibited the lowest exposure on study day 0 when compared to all other dogs. Its *C*_max_ was 2.8 times lower than the average, and its AUC was 1.8 times lower than the average. Furthermore, the exposure was also lower than the average expected values in fasted conditions. Conversely, on study day 13, this same dog displayed the highest exposure, altogether resulting in high *C*_max_ and AUC ratios. The high accumulation ratio calculated for this dog was deemed an isolated incident. With the exception of this dog, the accumulation ratios based on AUC were as follows: one dog had an accumulation ratio above 1 (*R* = 1.67), another dog had an accumulation ratio close to 1 (*R* = 1.1), and two dogs had ratios below 1 (*R* = 0.73 and 0.84).

**TABLE 3:** Summary of PK parameters of ilunocitinib following single or daily administrations of the final tablet formulation at 0.8 mg/kg in fasted dogs.

Variable (units)	Oral tablet	
	Day 0 0.8 mg/kg	Day 13 0.8 mg/kg
	Geometric mean (CV%)^a^ (*N* = 5)	
*T*_max_ (h)	3 (1–4)	1.50 (1.5–4)
*C*_max_ (ng/mL)	108 (83.5)	163 (60.9)
*C*_max_/D (ng/mL)	129 (75.0)	187 (61.0)
AUC_0‐24_ (h*ng/mL)	745 (43.0)	977 (57.0)
AUC_0‐24_/D (h*ng/mL)	894 (33.9)	1120 (58.7)
T_1/2_ (h)	4.29 (51.8)	3.97 (35.2)
R_acc_ C_max_/D^b^	—	1.45
R_acc_ AUC_0‐24_/D^b^	—	1.26

^a^ Geometric mean (and geometric CV%) except for Tmax where the median and (range) is given.

^b^ Accumulation ratios were calculated for each individual dog, and the geometric mean of the ratio is displayed.

### 3.4 Pharmacokinetics in Field Conditions

Visual inspection of ilunocitinib plasma concentrations measured at the different study days/visits, reflecting steady‐state levels given its relatively short half‐life, showed no visible time‐dependent differences across study visits.

**FIGURE 5:**
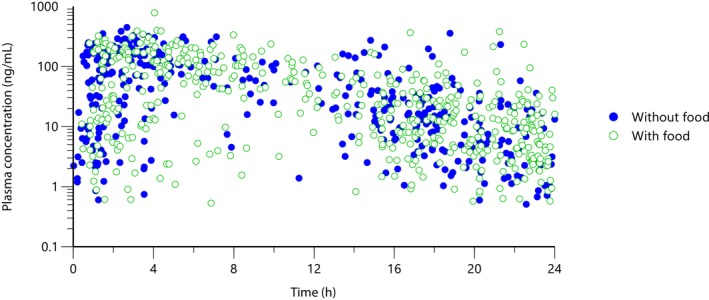
Plasma concentrations of ilunocitinib (0.6–0.8 mg/kg) in the clinical trials, with or without food.

The proportions of doses administered with and without food were as follows: Clinical Study 1 (68% with food, 32% without food); Clinical Study 2 (63% with food, 37% without food); and Clinical Study 3 (46% with food, 54% without food). While ilunocitinib bioavailability is known to be affected by prandial state under laboratory conditions, no significant differences in plasma concentrations were observed between fed and fasted states in the field (Figure 5). This correlated with consistent efficacy regardless of feeding status across all three trials.

## 4 Discussion

Ilunocitinib was well‐tolerated across all pharmacokinetic laboratory studies, with no serious adverse events reported. Minor adverse events, such as vomiting or diarrhea, were infrequent and transient.

Following intravenous administration, ilunocitinib exhibited a relatively low plasma clearance of 0.437 L/h/kg (7.3 mL/min/kg) and a terminal half‐life of approximately 4–5 h with a moderate volume of distribution of 1.58 L/kg.

The volume of distribution > 1 L/kg suggests distribution outside the plasma compartment into tissues and organs. This observation is consistent with a separate study where [^14^C]‐ilunocitinib was shown to be rapidly and widely distributed into numerous tissues in Beagle dogs (unpublished Elanco internal data). Blood–brain barrier penetration was low. High amounts of radioactivity were found in the bile, liver, and kidney. By 48 h post‐administration in dogs, approximately 80% of ilunocitinib was recovered: approximately 50% of ilunocitinib was eliminated via the biliary/fecal route, and 30% was excreted in urine.

Oral administration resulted in a *T*_max_ of approximately 1 to 4 h, consistent with the rapid onset of action observed as a significant decline in the mean Pruritus Visual Analog Scale (PVAS) score after the first dose (Forster, Trout, et al. 2025b).

To provide additional insights into the oral bioavailability, the in vitro permeability of ilunocitinib was experimentally determined in a Caco‐2 cell monolayer study (unpublished Elanco internal data). Ilunocitinib was tested at different concentrations, and the permeability coefficients (*P*_app_) for ilunocitinib indicated a moderate to high permeability (ranging from 6.74 × 10^–6^ cm/s to 17.45 × 10^–6^ cm/s). In the bi‐directional experiments, the efflux ratio ranged from 3.1 to 6.3, and the addition of verapamil decreased the efflux ratio, indicating that ilunocitinib is a substrate of gastrointestinal efflux transporter. Baricitinib, a close analog of ilunocitinib, and tofacitinib were shown to be P‐glycoprotein (P‐gp) substrates (Payne et al. 2015; EMA 2013), which could potentially reduce oral absorption. However, due to high permeability at higher concentrations and good oral bioavailability, potential P‐gp activity does not appear to significantly impact ilunocitinib absorption.

Since feeding can influence pharmacokinetics and may improve owner compliance, the impact of food on ilunocitinib absorption was evaluated. A laboratory bioavailability study revealed higher drug exposure (*C*_max_ and AUC) under fed conditions. Subsequently, the target animal safety study (Kuntz et al. 2025) was conducted under fed conditions, revealing no specific safety concerns. However, in field trials where owners administered tablets with or without food, consistent efficacy was observed regardless of feeding status. Although a formal laboratory pharmacokinetic study showed that food increases ilunocitinib bioavailability, pivotal field efficacy trials demonstrated a consistent clinical outcome regardless of feeding status. The laboratory studies mimicked the two extremes regarding prandial state (fasted for at least 16 h vs. fed maximal 30 min before treatment), whereas the situation in real life is distributed between these extremes. Consequently, the magnitude of the food effect is likely to be less pronounced under field conditions and therefore is not clinically significant, as the drug exposure achieved even in the fasted state is sufficient for therapeutic efficacy. We acknowledge that these field trials were not designed to assess pharmacokinetics, and the inherent variability of a clinical setting could obscure such effects. Ultimately, the consistent efficacy supports the flexibility of administering ilunocitinib without regard to meals.

Two studies, adhering to EMA Guideline (EMA 2024), assessed dose proportionality using three (0.8, 2.4, and 4.0 mg/kg; fed) and five (0.4, 0.8, 1.6, 2.4, and 4.0 mg/kg; fasted) dose levels. While dose linearity is important for products with variable dosing, ilunocitinib's fixed‐dose regimen meant these studies primarily aimed to characterize the pharmacokinetic profile at multiples of the therapeutic dose (0.8 mg/kg) to inform and de‐risk the pivotal target animal safety study. This approach, covering a 10‐fold range (0.4–4.0 mg/kg; 0.5× to 5× the therapeutic dose), provided a comprehensive assessment of dose effects. These dose effects have been confirmed in the pivotal target animal safety study (Kuntz et al. 2025), which used the final tablet formulation administered orally to fed dogs for 182 days at 1×, 2×, 3×, and 5× the therapeutic dose. Exposure (AUC_0‐24_ and C_max_) increased with dose, demonstrating a slightly less than dose‐proportional relationship. The observed less‐than‐dose‐proportional increase in exposure with increasing doses suggests a saturable process in the drug's absorption phase. The most likely cause is solubility‐limited absorption, stemming from ilunocitinib's low aqueous solubility, where at higher doses, the drug's dissolution in the gastrointestinal tract becomes the rate‐limiting step. Altogether, these findings at clinically relevant multiples of the intended dose, informed the safety margin established in the target animal safety study.

Ilunocitinib's terminal half‐life of approximately 5 h suggests minimal accumulation potential, supported by both theoretical considerations and observed data. From a theoretical perspective, for a drug administered daily with a terminal half‐life shorter than 12 h, the accumulation ratio will be less than 1.3 (Toutain and Bousquet‐Mélou 2004). Data from the repeat‐dose study (0.8 mg/kg/day) confirmed minimal accumulation in most dogs. The pivotal 182‐day target animal safety study, with doses at 1×, 2×, 3×, and 5× the therapeutic dose, showed only slight increases in exposure with increasing dose multiples (AUC ratios at study day 182 vs. study day 1: 1.2, 1.4, 1.6, and 1.2, respectively) (Kuntz et al. 2025). These findings confirm minimal accumulation with long‐term daily dosing. Therefore, based on study data and considering the terminal half‐life of ilunocitinib, there is no clinically relevant potential for continued accumulation and a maximum duration of administration does not need to be specified.

## 5 Conclusion

In conclusion, these studies characterized the pharmacokinetic profile of ilunocitinib in dogs, with properties suitable for once‐daily oral dosing. Ilunocitinib was rapidly absorbed after oral administration and displayed good oral bioavailability. The prandial effect observed in laboratory studies was not clinically relevant under field conditions. Confirmation of minimal accumulation in long‐term studies further supports the safety of chronic ilunocitinib administration.

## Author Contributions

Kirsten Boerngen: study design, analysis of the results, writing, review and editing. Yogini Patel: bioanalysis, writing, review and editing. Melissa Pittorino: study execution, writing, review and editing. Céline E. Toutain: project administration, analysis of the results, writing, review, and editing.

## Ethics Statement

The authors confirm that the ethical policies of the journal, as noted on the journal's author guidelines page, have been adhered to and the appropriate ethical review committee approval has been received. The authors confirm that they have adhered to either US or European standards for the protection of animals used for scientific purposes.

## Conflicts of Interest

All authors are current employees of Elanco Animal Health.

## Data Availability

Research data are not shared.
